# Formononetin reverses Treg/Th17 imbalance in immune-mediated bone marrow failure mice by regulating the PI3K/Akt signaling pathway

**DOI:** 10.1186/s13020-024-00919-9

**Published:** 2024-03-25

**Authors:** Huixuan Lan, Wei Qiu, Jie Wu, Zhijing Hu, Xiaomin Zhang, Lingling Zhu

**Affiliations:** 1 School of Traditional Chinese Medicine, Southern Medical University, Guangzhou, 510515 China; 2 Traditional Chinese Pharmacological Laboratory, School of Traditional Chinese Medicine, Southern Medical University, Guangzhou, 510515 China; 3 Department of Traditional Chinese Medicine, Nanfang Hospital, Southern Medical University, Guangzhou, 510515 China; 4 Department of Hematology, Hospital of Integrative Chinese and Western Medicine, Southern Medical University, Guangzhou, 510000 China; 5 Hospital of Integrative Chinese and Western Medicine, Southern Medical University, Guangzhou, 510000 China; 6 International Cancer Center, Shenzhen University Health Science Center, Shenzhen, 518060 China; 7 Department of Hematology and Oncology, Shenzhen University General Hospital, Shenzhen, 518060 China

**Keywords:** Formononetin, Treg/Th17 cells, Severe aplastic anemia, PI3K/Akt signaling pathway

## Abstract

**Background:**

Severe aplastic anemia (SAA) is a syndrome of bone marrow failure which is life-threatening. Recent studies have demonstrated that CD4 + T cell subsets, including T regulatory (Treg) and T helper 17 (Th17) cells, play a pivotal role in the pathogenesis of SAA. Formononetin (FMN) is a natural compound extracted from the traditional Chinese medicine Huangqi, which has the ability to regulate the imbalance of Treg/Th17 cells in some inflammatory diseases. Nevertheless, the therapeutic effect of FMN in SAA has yet to be definitively established. Therefore, the objective of this research was to investigate the effect of FMN on SAA and elucidate its underlying mechanism.

**Methods:**

In vivo experiments, the mice were divided into the following five groups: control, model, low-dose FMN, high-dose FMN, and positive control cyclosporine A group. The immune-mediated bone marrow failure (BMF) mouse model was established by the total body X-ray radiation and lymphocyte infusion. After 10 days of continuous administration of FMN, the numbers of Treg/Th17 cells in the bone marrow and spleen were assessed by flow cytometry. The protein expressions of PI3K/Akt pathway in the bone marrow and spleen was assessed by immunohistochemistry and western blotting. In vitro, the impact of FMN on the differentiation of naive CD4 + T cells into Treg cells was investigated by flow cytometry and ELISA.

**Results:**

In comparison with the control group, the model group showed a reduction in bone marrow nucleated cells, a significant decrease in peripheral blood cells, and an altered CD8 + /CD4 + T cell ratio. These findings indicate the successful establishment of a mouse model of immune-mediated BMF. After FMN treatment, there were the increased levels of red blood cells and hemoglobin. In addition, FMN mitigated the bone marrow destruction and restored the CD8 + /CD4 + T cell ratio. Furthermore, in comparison with the control group, the model group showed the decreased levels of Treg cells and the increased levels of Th17 cells. After FMN treatment, there was a significantly increased number of Treg cells and a decreased number of Th17 cells. Additionally, FMN remarkably down-regulated the expression levels of PI3K and Akt proteins in immune-mediated BMF mice.

**Conclusions:**

FMN alleviates immune-mediated BMF by modulating the balance of Treg/Th17 cells through the PI3K/Akt signaling pathway.

**Supplementary Information:**

The online version contains supplementary material available at 10.1186/s13020-024-00919-9.

## Introduction

Severe aplastic anemia (SAA) is a rare syndrome of bone marrow failure characterized by bone marrow hypoplasia and peripheral blood pancytopenia [1]. The primary pathogenesis of SAA is associated with immune-mediated bone marrow hematopoietic suppression, especially the cellular immune hyperfunction mediated by T lymphocytes [2, 3]. At present, the recommended therapeutic strategies for SAA mainly include hematopoietic stem cell transplantation and immunosuppressive therapy. Hematopoietic stem cell transplantation is challenging due to the difficulty in finding matching donors and the risk of immune rejection. Meanwhile, immunosuppressive therapy may not achieve the expected treatment outcome in some patients and may even lead to serious adverse reactions. Therefore, there is an urgent need to explore new treatment approaches for SAA with minimal side effects. However, due to the number of hematopoietic cells in the bone marrow of SAA patients is remarkably downregulated and the patient compliance is low, it is difficult to explore the mechanism through bone marrow in humans, which has formed a major obstacle for fully studying the pathogenesis of SAA. Previous studies have demonstrated that the pathogenesis of the immune-mediated BMF mouse model is similar to that of SAA patients [4, 5]. Thus, the immune-mediated BMF mouse model can be used to explore the pathogenesis of SAA and alternative therapies.

The CD8 + /CD4 + T cell ratio was increased in SAA patients, especially the increased number and frequency of CD8 + T cells, which may inhibit bone marrow hematopoiesis through direct cell–cell interactions [6, 7]. In addition, recent research has demonstrated that CD4 + T cell subsets, including T regulatory (Treg) and T helper 17 (Th17) cells, assume a crucial role in the pathogenesis of SAA [8–10]. Treg cells, which are an anti-inflammatory subpopulation of CD4 + T cells, exert immunomodulatory effects by releasing anti-inflammatory cytokines, such as IL-10 and TGF-β. On the contrary, Th17 cells are a pro-inflammatory subpopulation of CD4 + T cells, which could release IL-17, IL-22 and IL-23. There are increasing evidences indicating that the balance between Treg cells and Th17 cells assumes a crucial role in the maintenance of immune homeostasis [11]. In patients with SAA, the immunomodulatory activity of Treg cells is impaired, whereas Th17 cells are excessively activated [12, 13]. Thus, restoring the balance of Treg/Th17 cells can mitigate immune hyperactivity in SAA patients.

Previous studies have demonstrated that traditional Chinese herbs and the natural compounds extracted from them exhibit immunotherapeutic efficacy in BMF-related diseases with the advantages of low toxicities [14–16]. Huangqi, also named *Astragalus membranaceus*, is one of the main components in many herbal formulas and extensively utilized in the treatment of a variety of diseases [17, 18]. It is well established that Huangqi possesses excellent immunomodulatory and anti-inflammatory activities [19, 20]. Formononetin (FMN) is a natural isoflavone compound extracted from the traditional Chinese medicine Huangqi. Modern pharmacological studies have reported that FMN possesses anti-inflammatory, antimicrobial, antioxidant, anticancer and immunomodulatory properties [21]. It has been confirmed that Huangqi could regulate the balance of Treg/Th17 cells in inflammatory diseases, such as asthma and colitis [22–24]. FMN could modulate the differentiation of Th17 cells and facilitate the development of Treg cells in mice with estrogen deficiency-induced osteoporosis and consequently alleviate bone loss [25]. However, the effect of FMN on the proportion of Treg/Th17 cells in immune-mediated BMF mice remains unknown. Therefore, it is reasonable to speculate whether FMN can play a therapeutic role in SAA by regulating the Treg/Th17 cell ratio. This study demonstrated that FMN mitigates immune-mediated BMF by regulating the proportions of T cell subsets, particularly the Treg/Th17 cell ratio. Furthermore, this study suggested that the potential therapeutic mechanism of FMN involved the PI3K/Akt pathway, which provides novel insights for the therapeutic application of traditional Chinese medicine in SAA.

## Materials and methods

### Reagents and materials

Formononetin (purity ≥ 98.0%) was purchased from Yuanye (Shanghai, China). TNF-α (430904), IFN-γ (430804) and IL-12 (431005) ELISA kits and all flow antibodies were obtained from BioLegend (San Diego, CA, USA). The naive CD4 + T cell isolation (130-104-453) was purchased from Miltenyi Biotec (Auburn CA). anti-IL-4 (504122), anti-IFN-γ (505834), anti-CD28mAb (102116),anti-CD3mAb (100340),anti-CD4-FITC (116004), anti-CD8-PE/Dazzle™ 594 (100762), anti-CD3-APC (100236), anti-CD4-FITC (100510), anti-CD25-Brilliant Violet 421™ (102033), anti-Foxp3-Alexa Fluor 647 (320014) and anti-IL-17a-PE (506903) antibodies were obtained from BioLegend (San Diego, CA). mIL-2 (200-02-10) and hTGF-β1 (100-21) were obtained from PeproTech (USA). p-PI3K (4228 T), PI3K (4249 T), p-Akt (4060 T), Akt (4691 T), Foxp3 (14-5773-80) and β-Actin (8475S) antibodies were obtained from Cell Signaling Technology (Danvers, MA, USA) and Invitrogen (Carlsbad, CA, USA).

### Animals

Female CByB6F1 mice (bodyweight: 21–26 g) as the experimental models was obtained from Beijing Vital River Laboratory Animal Technology Co., Ltd. Donor C57BL/6J mice was obtained from the Guangdong Medical Laboratory Animal Center.

### Animal grouping and immune-mediated BMF model establishment

CByB6F1 mice (n = 30) were divided into the following five groups (6 mice/group): control, model, cyclosporine A (CsA), low-dose FMN (FMN-L), and high-dose FMN (FMN-H) groups. The thymus and lymph nodes were collected from donor C57BL/6J mice, homogenized, centrifuged, and resuspended to obtain the lymphocyte suspension by combining thymocytes and lymph node cells at a ratio of 1:2. Except for the control group, all other groups were established by the total body X-ray radiation, followed by intravenous injection of 0.2 mL of the lymphocyte suspension [26]. Drug administration started 24 h after modeling. Mice in the FMN-L or FMN-H group were orally at a dosage of 50 mg/kg or 100 mg/kg bodyweight/day FMN. Meanwhile, mice in the CsA group were intraperitoneally administered with CsA (25 mg/kg bodyweight/day). Mice in the control and model groups were orally administered with an equivalent volume of physiological saline. After 10th day of drug administration, the mice were euthanized to collect samples for further analysis.

### Peripheral blood cell analysis

After 10th day of drug administration, peripheral blood samples were collected in EDTA anticoagulant tubes by enucleation of the eyeballs. The peripheral blood sample (100 μL) was subjected to automated hematology analysis to quantify the level of white blood cells (WBC), red blood cells (RBC), hemoglobin (HGB), and platelets (PLT).

### Histopathological analysis

The sternum was dissected and fixed with 4% paraformaldehyde for 48 h. The femur was softened using and decalcifying solution, dehydrated using a gradient ethanol series, embedded in paraffin, sectioned, and stained with hematoxylin and eosin. The histopathological changes in each group were observed under an optical microscope. The number of nucleated cells and megakaryocytes in three random high-power fields (400 ×) of each slide was counted and take the average result for statistical analysis.

### Flow cytometry analysis

The bone marrow and spleen cells were obtained from the mice and subjected to RBC, counting and processed to obtain the cell suspension. The cells were divided into three groups. The cells in the first group were incubated with Anti-CD4-FITC, anti-CD8-PE/Dazzle™ 594 and anti-CD3-APC antibodies for 30 min and fixed to detect the allocation of T cell subsets. The cells in the second group were incubated with anti-CD4-FITC and anti-CD25-Brilliant Violet 421™ antibodies, fixed, permeabilized, and stained with anti-Foxp3-Alexa Fluor 647 antibodies for 30 min to detect the percentage of Treg cells. Meanwhile, the cells in the third group were stimulated with a stimulant comprising phorbol myristate acetate, ionomycin, and monensin for 5 h, incubated with anti-CD4-FITC antibodies for a duration of 30 min, fixed, permeabilized, and stained with anti-IL-17a-PE antibodies to detect thepercentage of Th17 cells. A single antibody-positive tube was used for compensation adjustment. Flow cytometric analysis was performed using a FACSCalibur instrument, following the manufacturer’s instructions.

### Naive CD4 + T cell sorting and polarization

The spleen and lymph nodes were isolated from C57BL/6J mice, homogenized, centrifuged, and resuspended to prepare the lymphocyte suspension. Naive CD4 + T cells were isolated by magnetic-activated cell sorting (MACS) in accordance with the manufacturer’s instructions from lymphocyte suspensions. Then, the naive CD4 + T cells were cultured in a 24-well plate at 4 × 10^5^ cells per well, and 10 ng/mL mIL-2, 10 ng/mL hTGF-β1, 5 µg/mL anti-IL-4, 5 µg/mL anti-IFN-γ and 2.5 µg/mL anti-CD28mAb were added to plates coated with 5 µg/mL anti-CD3mAb to promote the differentiation of Treg cells. After 3 days, Treg cell polarization was examined.

### Serum analysis using enzyme-linked immunosorbent assay (ELISA)

The serum levels of IL-12, TNF-α, and IFN-γ in the immune-mediated BMF mice and the level of IL-10 in the cell supernatant were assessed by ELISA. The concentration of these cytokines was determined following the manufacturer’s instructions.

### Western blotting

The remaining spleen cells were lysed using RIPA lysis buffer containing protease and phosphatase inhibitors. The sample lysates were, vortexed, and centrifuged. The protein concentrations were determined using the BCA protein quantification kit. The proteins were denatured using 5 × SDS buffer. Equal amounts (40 μg) of proteins were subjected to SDS–polyacrylamide gel electrophoresis. The resolved proteins were transferred to a PVDF membrane. The membranes were blocked with 5% non-fat dry milk in Tris-buffered saline containing Tween-20 (TBST) at room temperature for 2 h and incubated with the p-PI3K, PI3K, p-Akt, Akt, Foxp3 and β-Actin (1:1000) antibodies overnight at a 4 °C. After washing with TBST, the membranes were incubated with the secondary antibodies at room temperature for 1 h. The membranes were washed again with TBST. Immunoreactive bands were detected using ECL luminescence reagent. The grayscale values of the protein bands were determined using ImageJ software.

### Immunohistochemistry analysis

The paraffin sections were dewaxed, dehydrated, and subjected to antigen retrieval. To block endogenous peroxidase, the sections were incubated with 3% hydrogen peroxide. The sections were incubated with BSA to block nonspecific binding. Next, the sections were placed in a humid chamber at 4 °C and incubated overnight with PI3K, Akt, and Foxp3 primary antibodies (all 1:250). All antibodies were obtained from Cell Signaling Technology (Danvers, MA, USA) and Invitrogen (Carlsbad, CA, USA). After washing with phosphate-buffered saline, the sections were incubated with secondary antibodies at room temperature for 30 min. Immunoreactive signals were detected using diaminobenzidine (DAB), and the sections were counterstained with hematoxylin. The sections were dehydrated and mounted on glass slides for microscopic analysis. The protein expression levels were independently evaluated by two researchers. The number of Foxp3-positive cells in three random high-power fields (400 ×) of each slide was counted and take the average result for statistical analysis. PI3K and Akt were mainly localized to the cytoplasm. The immunofluorescence intensities of PI3K and Akt were semi-quantitatively analyzed using ImageJ software. The sections without primary antibody but incubated with the same concentration of secondary antibody were used as the negative control.

### Statistical analysis

All statistical analyses were performed using SPSS 26.0 software. The experimental data are presented as mean ± standard error of mean. Means between groups were compared using one-way analysis of variance for normally distributed data or nonparametric tests for non-normally distributed data. Graphing was performed using GraphPad Prism 9 software. *p* < 0.05 was considered as a statistically significant difference.

## Result

### FMN alleviates immune-mediated BMF

**Fig. 1:**
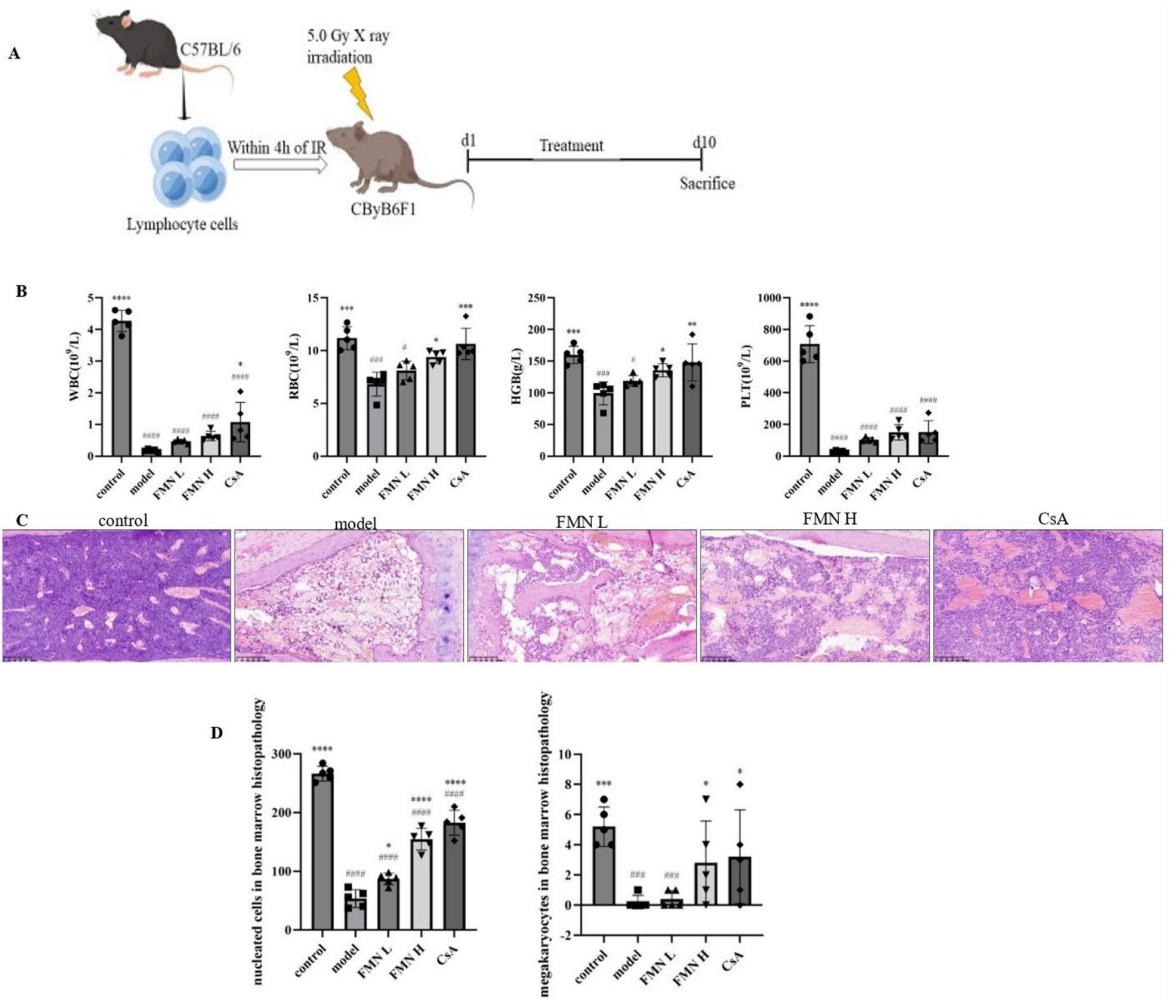
HYPERLINK "sps:id::fig1||locator::gr1||MediaObject::0" Effect of FMN in the immune-mediated BMF mouse model. **A** The establishment of the immune-mediated BMF mouse model and the treatment process. **B** The levels of white blood cells (WBC), red blood cells (RBC), hemoglobin (HGB), and platelets (PLT) in the peripheral blood of different groups (n = 5). **C** Bone marrow pathological characteristics in different groups were examined by hematoxylin and eosin staining (200 ×) (n = 5). **D** The number of nucleated cells and megakaryocytes of bone marrow pathology in different groups (400 ×) (n = 5). *p < 0.05, **p < 0.01, ***p < 0.001, ****p < 0.0001 compared with the model group. #*p* < 0.05, ###*p* < 0.001, ####*p* < 0.0001 compared with the control group

In order to evaluate the therapeutic effect of FMN on SAA, the immune-mediated BMF mouse model, which has been validated as a suitable model for evaluating the pathogenesis of SAA, was established by infusing the sublethally irradiated recipient CByB6F1 mice with lymphocytes from C57BL/6J donors (Fig. 1A). Then, the levels of peripheral blood, including WBC, RBC, PLT and HGB were quantified. The levels of peripheral blood cells in the model group were significantly lower than those in the control group. Compared with the model group, The levels of RBC and HGB were remarkably increased in the FMN-H groups (Fig. 1B). Pathological examination showed that the bone marrow contained numerous nucleated cells and megakaryocytes in the control group, while the bone marrow of the model group exhibited the decreased number of nucleated cells and a lack of megakaryocytes accompanied with the increased number of adipocytes. Compared with the model group, the number of nucleated cells and megakaryocytes was significantly increased, which indicates that FMN promotes bone marrow hematopoiesis in the immune-mediated BMF mice (Fig. 1C).

### FMN restores the balance of CD8 + /CD4 + T cells and Treg/Th17 cells in the immune-mediated BMF mice

**Fig. 2:**
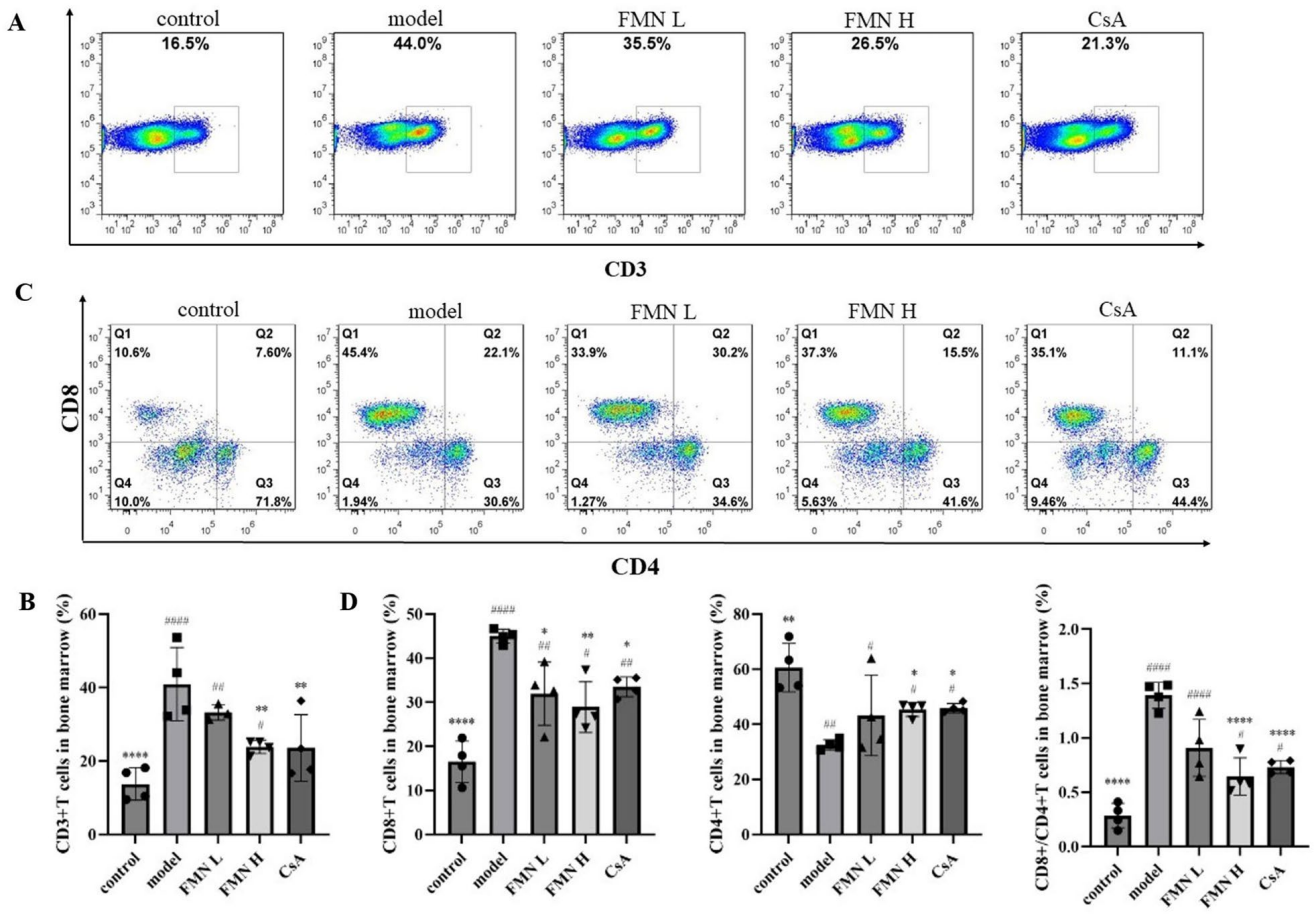
Effect of FMN on the proportion of T cells in the immune-mediated BMF mouse model. **A**–**D** The proportion of CD3 + T cells, CD4 + T cells and CD8 + T cells in different groups was examined by flow cytometry (n = 4). *p < 0.05, **p < 0.01, ****p < 0.0001 compared with the model group. #*p* < 0.05, ##*p* < 0.01, ####*p* < 0.0001 compared with the control group

The pathogenesis of SAA is the destruction of bone marrow hematopoietic cells by aberrantly activated T lymphocytes [1]. Thus, the percentages of CD3 + T cells, CD4 + T cells and CD8 + T cells were measured in the immune-mediated BMF mice in the present study. Compared with the control group, the proportions of CD3 + T cells and CD8 + T cells as well as the ratio of CD8 + / CD4 + T cells were upregulated in the model group, while the proportion of CD4 + T cells was decreased. Interestingly, after treatment with FMN and CsA, the percentages of CD3 + T cells, CD8 + T cells and CD8 + /CD4 + T cell ratio were decreased in the immune-mediated BMF mice, while the percentage of CD4 + T cells was upregulated (Fig. 2A–D). These indicate that FMN could decrease the levels of T lymphocytes and regulate the balance of CD8 + /CD4 + T cell subsets in immune-mediated BMF mice, which showed similar efficacies with the positive control drug, CsA.

**Fig. 3:**
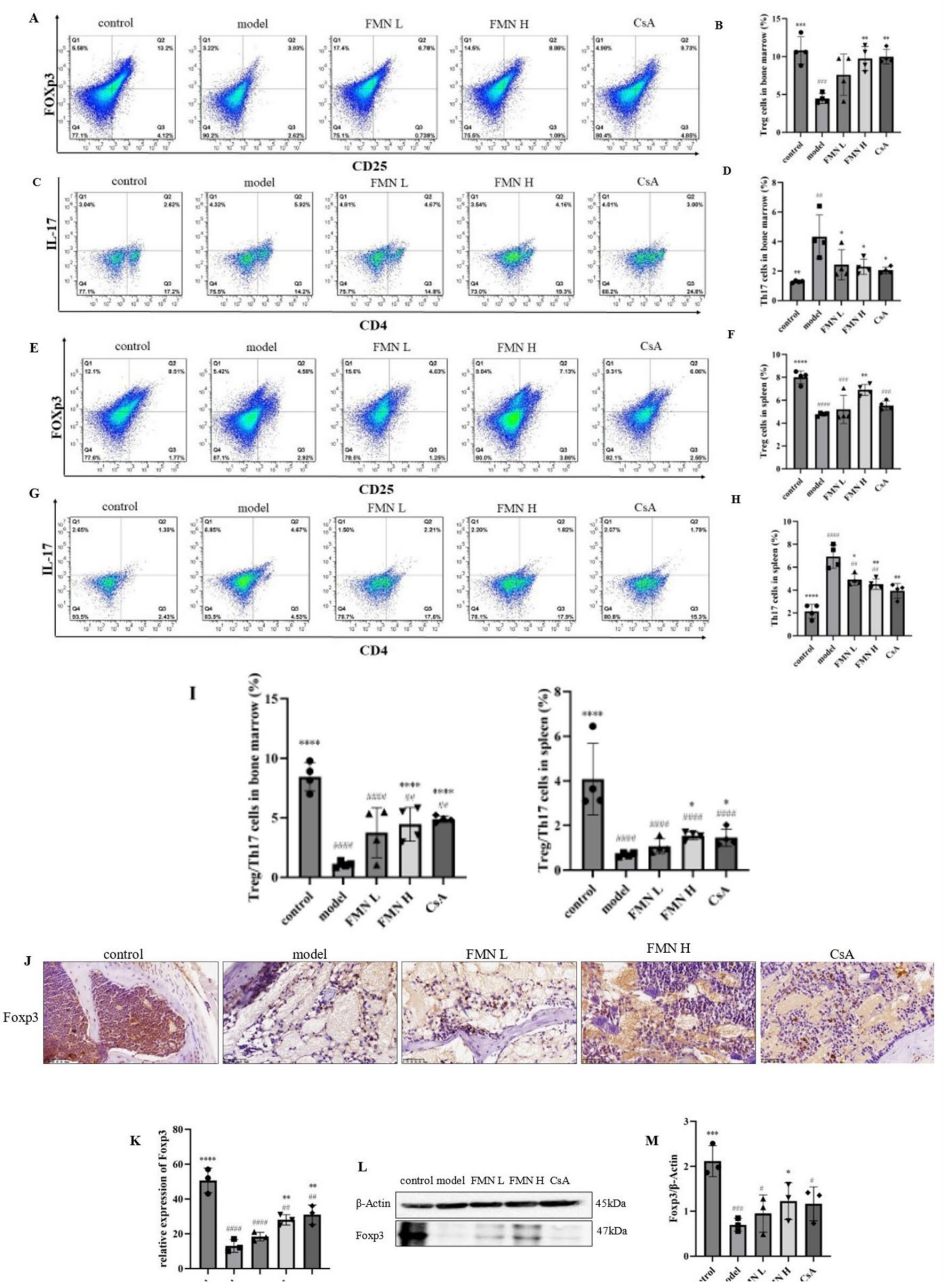
Effects of FMN on the proportion of Treg cells and Th17 cells in the bone marrow and spleen of the immune-mediated BMF mice (n = 4). **A**, **B** The proportion of Treg cells in the bone marrow was assessed using flow cytometry. **C**, **D** The proportion of Th17 cells in the bone marrow was assessed using flow cytometry. **E**, **F** The proportion of Treg cells in the spleen was evaluated using flow cytometry. **G**, **H** The proportion of Th17 cells in the spleen was evaluated using flow cytometry. **I** The ratio of Treg/Th17 cells in in the bone marrow and spleen of the immune-mediated BMF mice. **J**, **K** The expression levels of Foxp3 in the bone marrow were determined using immunohistochemical analysis (400 ×). **L**, **M**: The expression levels of Foxp3 in the spleen were determined by western blotting. *p < 0.05, **p < 0.01, ***p < 0.001, ****p < 0.0001 compared with the model group. #*p* < 0.05, ##*p* < 0.01, ###*p* < 0.001, ####*p* < 0.0001 compared with the control group

In addition, we further explored the effects of FMN on the balance of Treg/Th17 cells in immune-mediated BMF mice. Compared with the control group, the levels of Treg cells and the ratio of Treg/ Th17 cells in bone marrow and spleen of the model group were downregulated, whereas the levels of Th17 cells in bone marrow and spleen of the model group were markedly upregulated. After treatment with FMN and CsA, the percentage of Treg cells as well as the ratio of Treg/Th17 cells were increased, while the percentage of Th17 cells was downregulated (Fig. 3A–I). Furthermore, the expression of the transcription factor Foxp3 in Treg cells of immune-mediated BMF mice was examined by immunohistochemical analysis and western blotting. Compared with the control group, The expression levels of Foxp3 in the bone marrow and spleen of the model group were markedly downregulated. After treatment with FMN, the expression levels of Foxp3 were significantly upregulated (Fig. 3J–M). These findings suggest that FMN could reverse the imbalance of Treg/Th17 cells in the immune-mediated BMF mice.

### FMN downregulates inflammatory cytokines in the immune-mediated BMF mice

**Fig. 4:**
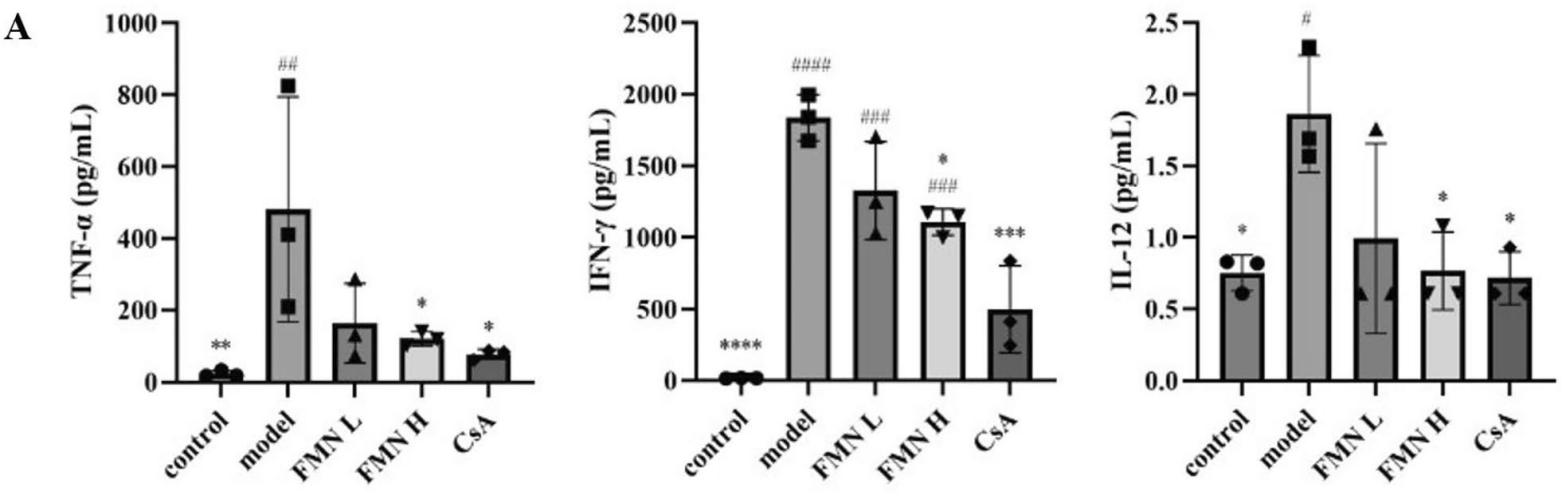
Effect of FMN on the levels of inflammatory cytokines in immune-mediated BMF mice. **A** The serum levels of TNF-α, IIFN-γ and IL-12 in different groups were evaluated using enzyme-linked immunosorbent assay (n = 3). *p < 0.05, **p < 0.01, ***p < 0.001, ****p < 0.0001 compared with the model group. #*p* < 0.05, ##*p* < 0.01, ###*p* < 0.001, ####*p* < 0.0001 compared with the control group

To explore the anti-inflammatory effects of FMN, the levels of inflammatory cytokines TNF-α, IL-12, and IFN-γ in serum were determined by ELISA kits. Compared with the control group, the levels of TNF-α, IL-12, and IFN-γ were significantly upregulated in the model group. After treatment with FMN-H, the levels of TNF-α, IL-12, and IFN-γ were markedly downregulated in immune-mediated BMF mice. These indicates that FMN exerts potent anti-inflammatory effects in the immune-mediated BMF mice (Fig. 4A).

### FMN promotes the differentiation of naive CD4 + T cells into Treg cells in vitro

**Fig. 5:**
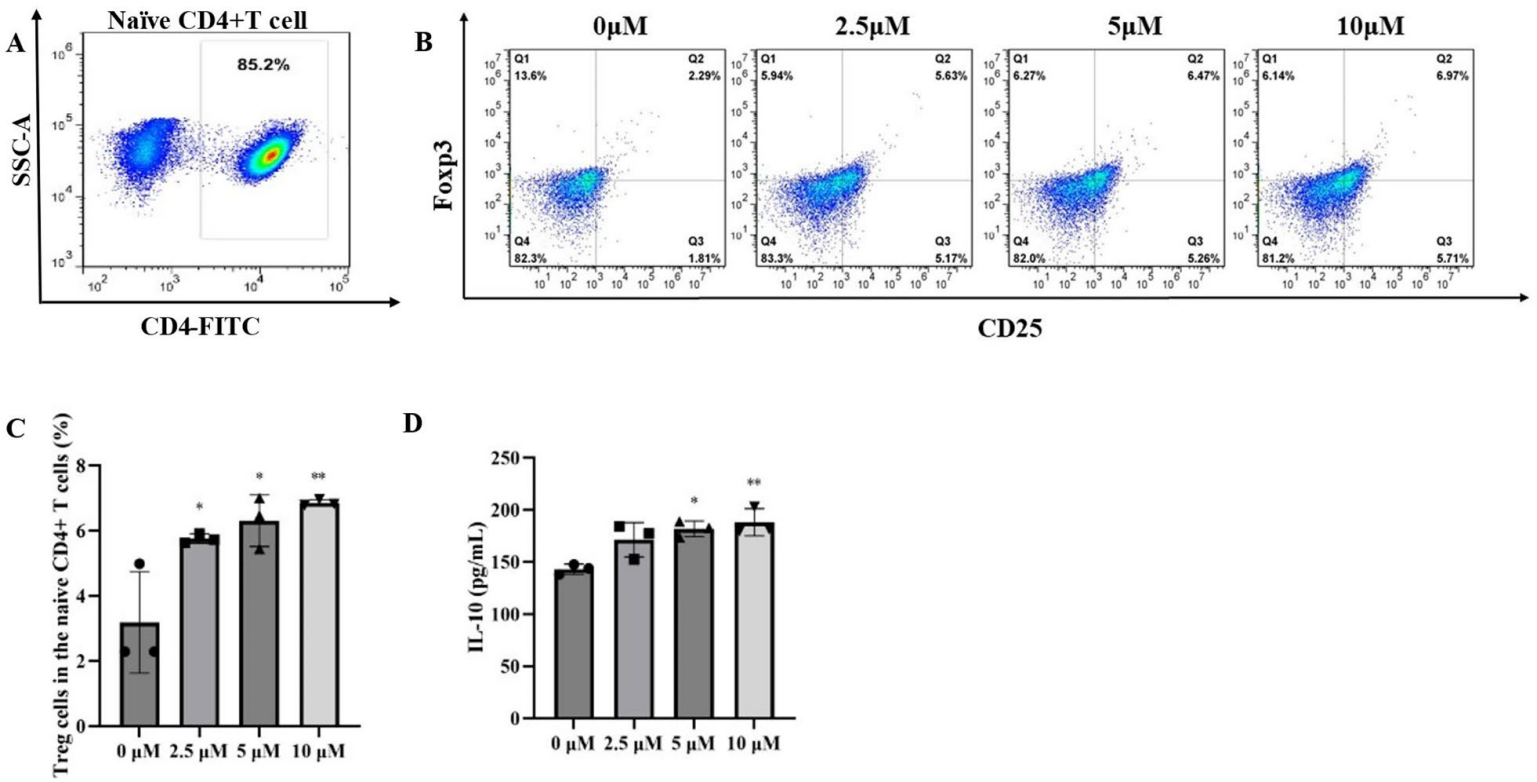
Effect of FMN on the differentiation of naive CD4 + T cells into Treg cells in vitro. **A** The purity of CD4 + T cells was determined using MACS. **B**, **C**: FMN promotes the differentiation of naive CD4 + T cells into Treg cells in vitro. **D** FMN promotes the production of IL-10, a representative cytokine secreted by Treg cells. *p < 0.05, **p < 0.01 compared with the model group

In vitro, the effect of FMN on the differentiation of naive CD4 + T cells into Treg cells was also investigated. The purity of the separated naive CD4 + T cells, as determined using flow cytometry, was > 85% (Fig. 5A). Firstly, the effect of FMN on naive CD4 + T cell viability was examined using the CCK-8 assay. We found that FMN did not adversely affect naive CD4 + T cell viability at concentrations less than 10 μM (Additional file 1: Fig S1). According to these results, the concentrations of 2.5, 5, and 10 μM FMN were selected for follow-up experiments. Three days after administration of FMN, flow cytometry was performed to assess the differentiation of naive CD4 + T cells into Treg cells. Compared with 0 μM FMN group, the proportions of Treg cells were markedly upregulated in 2.5 μM FMN, 5 μM FMN, and 10 μM FMN groups, and 10 μM FMN group exhibited the highest level of Treg cells (Fig. 5B, C). Furthermore, the levels of IL-10 secreted by Treg cells were assessed. Compared with 0 μM FMN group, the levels of IL-10 were markedly upregulated in 5 μM FMN and 10 μM FMN groups (Fig. 5D). These results indicate that FMN could promote the differentiation of naive CD4 + T cells into Treg cells.

### FMN mitigate immune-mediated BMF through the PI3K/Akt pathway

**Fig. 6:**
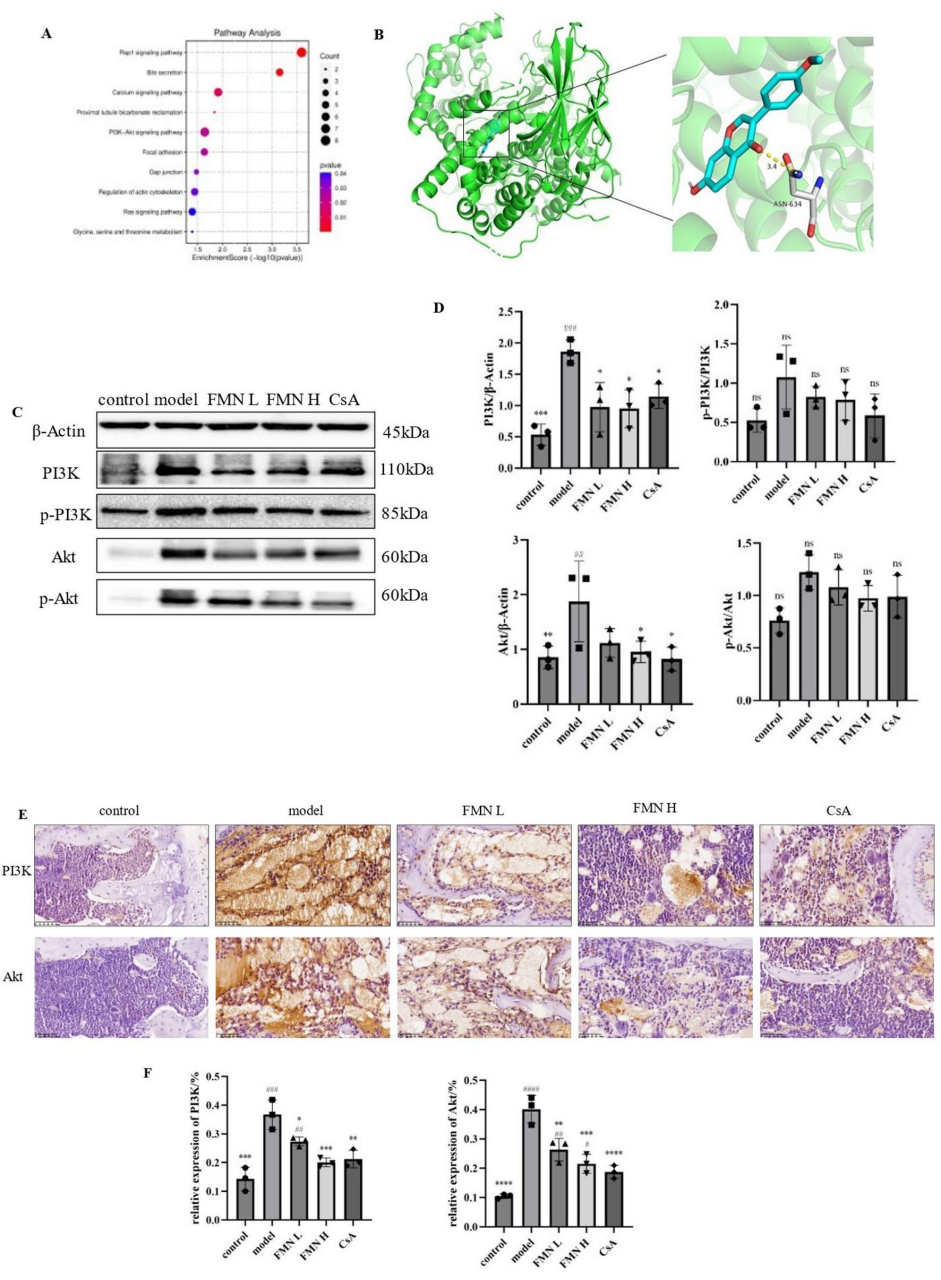
Effect of FMN on the PI3K/Akt signaling pathway in the immune-mediated BMF mouse model (n = 3). **A** Cluster analysis of GEO datasets. **B**: Molecular docking of FMN with PI3K. **C**, **D**: The expression levels of PI3K, p-PI3K, Akt, and p-Akt in different groups were evaluated using western blotting. **E**, **F** The expression levels of PI3K and Akt in bone marrow were detected by immunohistochemical analysis (400 ×). *p < 0.05, **p < 0.01, ***p < 0.001, ****p < 0.0001 compared with the model group. #*p* < 0.05, ##*p* < 0.01, ###*p* < 0.001, ####*p* < 0.0001 compared with the control group

The molecular mechanism by which FMN alleviates immune-mediated BMF was examined. Microarray based gene expression profiling (GSE3807) revealed that the PI3K/Akt pathway was significantly enriched among SAA patients (Fig. 6A). Molecular docking analysis suggested that FMN directly interacted with PI3K (Fig. 6B). These findings suggested that FMN may exert therapeutic effects on SAA through the PI3K/Akt pathway. Firstly, the expression levels of PI3K, p-PI3K, Akt, and p-Akt in the spleen of immune-mediated BMF mice were determined by western blotting. Compared with the control group, the expression levels of PI3K and Akt in the model group were significantly upregulated. The expression levels of PI3K and Akt in the spleen of immune-mediated BMF mice were significantly downregulated after treatment with FMN (Fig. 6C, D). Furthermore, the expression levels of PI3K and Akt in the bone marrow were examined by immunohistochemical analysis. Compared with the control group, the expression levels of PI3K and Akt were significantly upregulated in the model group. After treatment by FMN, the expression levels of PI3K and Akt in the bone marrow of immune-mediated BMF mice were significantly downregulated (Fig. 6E, F). Therefore, the expression levels of PI3K and Akt in the bone marrow of immune-mediated BMF mice were consistent with those in the spleen.

## Discussion

Currently, SAA is considered to be a bone marrow failure disorder induced by the hyperfunction of T lymphocytes, and the immune-mediated BMF mouse model has been validated as an animal model for investigating the potential pathogenesis of SAA and identifying novel therapeutic agents [27, 28]. Consistent with the previously reported literature, the mouse model of immune-mediated BMF was successfully established by combining 5.0 Gy total body irradiation with the infusion of 8*10^5 lymphocytes in CByB6F1 mice, mainly presented with the decreased peripheral blood cells and bone marrow cellularity, as well as an increased CD8 + /CD4 + T cell ratio. FMN, a bioactive component of Huangqi, possesses a variety of pharmacological activities, including anti-inflammatory and immunomodulatory activities [21]. In our present study, we evaluated the potential immunomodulatory effects of FMN on immune-mediated BMF. As expected, we demonstrate that FMN could effectively alleviate immune-mediated BMF through promoting hematopoiesis, protecting bone marrow nucleated cells, and restoring the balance of CD8 + /CD4 + T cells and Treg/Th17 cells, as well as down-regulating the levels of pro-inflammatory cytokines in immune-mediated BMF mice, including IFN-γ, TNF-α and IL-12. Similarly, FMN promoted the differentiation of naive CD4 + T cells into Treg cells in vitro. Moreover, our data indicate that the inhibition of PI3K/Akt pathway is essential for the therapeutic efficacy of FMN in immune-mediated BMF mice.

It’s well-known that aberrantly activated T cell-mediated destruction of hematopoietic cells is the main mechanism underlying the pathogenesis of SAA, especially CD8 + T cells [29]. In SAA patients, the activated CD8 + T cells is significantly upregulated, and the proportion of CD8 + /CD4 + T cells is inverted [29]. SAA patients exhibit a significantly higher ratio of CD8 + /CD4 + T cells, which is attributed to a diminished number of CD4 + T cells and a higher proportion of CD8 + T cells in bone marrow [30, 31]. More importantly, it has been reported that the over-activation of CD8 + T cells leads to the destruction of hematopoietic cells through Fas/Fas ligand (FasL) pathway and the release of negative regulators of hematopoiesis in SAA patients, such as IFN-γ, TNF-α [32]. Thus, inhibiting the over-activation of CD8 + T cells and restoring the ratio of CD8 + /CD4 + T cells may play a therapeutic role in SAA. The present study analyzed immunomodulatory effect of FMN in immune-mediated BMF mice. We found that FMN significantly decreased the proportion of T cells in bone marrow and reversed the imbalance of CD8 + /CD4 + T cells by downregulating the percentage of CD8 + T cells and upregulating the percentage of CD4 + T cells. Besides, high-dose FMN significantly downregulated the levels of inflammatory cytokines IFN-γ and TNF-α, which are mainly secreted by activated CD8 + T cells in SAA and involved in mediating the destruction of hematopoietic cells. These findings suggest that FMN could inhibit over-activation of CD8 + T cells and modulate the balance of CD8 + /CD4 + T cells in immune-mediated BMF mice, eventually mitigating immune-mediated destruction of hematopoietic cells.

CD4 + T cells are able to differentiate into various lineages of T helper (Th) cells, including Th1, Th2, Th17, and Treg cells [33]. In addition, multiple studies have demonstrated that in patients with SAA, the immunomodulatory activity and number of Treg cells are decreased, whereas Th17 cells are excessively activated, which suggests that there is an imbalance of Treg/Th17 cells in SAA [8, 34]. The imbalance of Treg/Th17 cells is associated with the increased severity of SAA, indicating that maintaining the balance of Treg/Th17 cells could also exert a therapeutic effect in SAA [12]. In the present study, we found that FMN could increase the proportion of Treg cells and decrease the percentage of Th17 cells. Additionally, FMN upregulated the expression levels of Foxp3 in the immune-mediated BMF mice, which is a lineage-determining transcription factor of Treg cells. Similarly, we also found that FMN promoted the differentiation of naive CD4 + T cells into Treg cells and upregulated the levels of IL-10 in vitro. These results indicate that FMN could also modulate the balance of Treg/Th17 cells in immune-mediated BMF mice, which is associated with regulating the differentiation of naive CD4 + T cells.

Our findings showed that FMN exerted the obvious therapeutic effects in immune-mediated BMF, and then the underlying mechanism was explored. The PI3K/Akt pathway is essential for proliferation, survival, and differentiation of hematopoietic cells [35, 36], as well as the proliferation and differentiation of CD4 + T cells after stimulation [37, 38]. At present, several studies have confirmed that the PI3K/Akt pathway is involved in the pathogenesis of SAA [19, 36]. Furthermore, molecular docking analysis showed that FMN could directly interacted with PI3K. Therefore, we further explored whether FMN could regulate the PI3K/Akt pathway in immune-mediated BMF mice. As expected, the expression levels of PI3K and Akt were distinctly downregulated after treatment with FMN. These findings suggested that FMN may exert therapeutic effects in SAA through inhibiting the PI3K/Akt pathway. However, there are several limitations in the present study. For example, there is a lack of PI3K inhibitors which can be utilized as the positive control in mechanistic studies.

## Conclusion

In summary, this study confirmed that FMN exerts therapeutic effects on immune-mediated BMF by promoting hematopoiesis and modulating T cell subset balance, including the balance of Treg/Th17 cells and CD8 + /CD4 + T cells, as well as inhibiting the production of inflammatory cytokines, like IFN-γ, TNF-α and IL-12. Additionally, the underlying therapeutic mechanism of FMN in immune-mediated BMF is involved in the suppression of the PI3K/Akt signaling pathway. Thus, FMN could be served as a supplementary choice for SAA treatment because of its immunomodulatory effects These findings also offer novel perspectives for the treatment of SAA with traditional Chinese medicine.

### Supplementary Information

**Additional file 1: Figure S1.** Effects of FMN on CD4 + T cell viability were evaluated using the CCK8 assay.

## Data Availability

The data that support the findings of this study are available from the corresponding author upon reasonable request.
