# Supplementary material for: Formononetin reverses Treg/Th17 imbalance in immune-mediated bone marrow failure mice by regulating the PI3K/Akt signaling pathway
Source: Chin Med. 2024 Mar 25;19:55. doi: 10.1186/s13020-024-00919-9 (PMC10962105; doi:10.1186/s13020-024-00919-9)
Supplement: Supplementary file 1 — Additional file 1: Figure S1. Effects of FMN on CD4 + T cell viability were evaluated using the CCK8 assay. [file 13020_2024_919_MOESM1_ESM.docx]

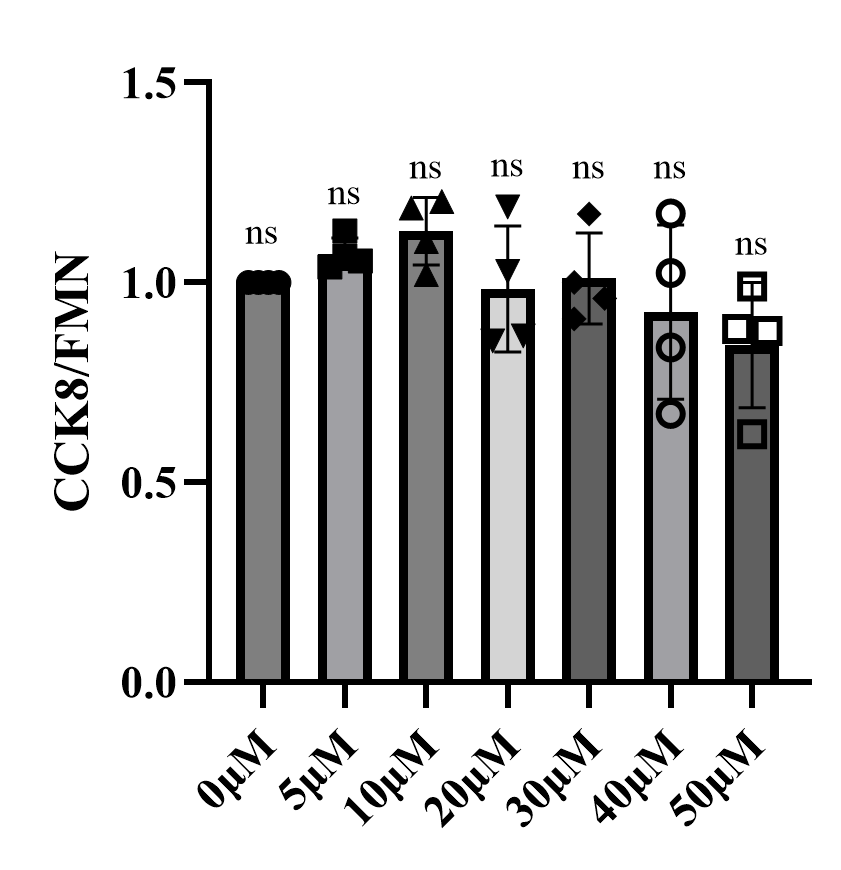


**Fig. S1** Effects of FMN on CD4+ T cell viability were evaluated using the CCK8 assay. ns, nonsignificant vs. 0μM.
